# Empagliflozin (an SGLT2 inhibitor), alone or in combination with linagliptin (a DPP-4 inhibitor), prevents steatohepatitis in a novel mouse model of non-alcoholic steatohepatitis and diabetes

**DOI:** 10.1186/s13098-016-0169-x

**Published:** 2016-07-26

**Authors:** Teruo Jojima, Takanori Tomotsune, Toshie Iijima, Kazumi Akimoto, Kunihiro Suzuki, Yoshimasa Aso

**Affiliations:** 1 Department of Endocrinology and Metabolism, Dokkyo Medical University, Mibu, Tochigi Japan; 2 Division of Clinical Science, Research Support Center, Dokkyo Medical University, Mibu, Tochigi Japan

**Keywords:** Non-alcoholic steatohepatitis, Non-alcoholic fatty liver disease, Dipeptidyl peptidase- 4, Sodium-glucose co-transporter 2, Inflammation, Fibrosis

## Abstract

**Background:**

Sodium-glucose co-transporter-2 (SGLT2) inhibitors are new oral antidiabetic drugs that reduce hyperglycemia by promoting urinary glucose excretion. Glycosuria produced by SGLT2 inhibitors is associated with weight loss, mainly due to reduced fat volume. We investigated the effects of empagliflozin (selective SGLT2 inhibitor) and linagliptin (DPP-4 inhibitor) on steatohepatitis and fibrosis in a mouse model of non-alcoholic steatohepatitis (NASH) with diabetes.

**Methods:**

A novel NASH model was generated by administration of streptozotocin to C57BL/6J mice at 2 days old, with a high-fat diet from 4 weeks. NASH mice aged 6 weeks were divided into four groups of 6 animals: vehicle, linagliptin (10 mg/kg), empagliflozin (10 mg/kg), and linagliptin + empagliflozin. The histological non-alcoholic fatty liver disease activity score was significantly lower in the empagliflozin and linagliptin + empagliflozin groups than in the vehicle or linagliptin groups. Hepatic expression of inflammatory genes (tumor necrosis factor-α, interleukin-6, and monocyte chemoattractant protein-1) was decreased in the empagliflozin and linagliptin + empagliflozin groups compared with the vehicle group. The collagen deposition with Sirius red staining was significantly reduced in the linagliptin + empagliflozin group compared with the linagliptin or the empagliflozin group. Immunohistochemistry showed that expression of α-smooth muscle actin, a marker of myofibroblasts (fibrosis), was reduced in the linagliptin + empagliflozin group compared with the vehicle group, as was expression of type 1 and 3 collagen mRNA. Linagliptin + empagliflozin decreased expression of mRNAs for genes related to fatty acid synthesis, but did not increase mRNAs for β-oxidation-related genes.

**Conclusions:**

While empagliflozin alone attenuates development of NASH showing anti-steatotic and anti-inflammatory effects, combined administration of empagliflozin and linagliptin can synergistically ameliorates NASH with stronger anti-fibrotic effects.

## Background

Non-alcoholic fatty liver disease (NAFLD) is now a common disease and has become a serious public health problem worldwide [1]. Up to 30 % of patients with NAFLD progress to non-alcoholic steatohepatitis (NASH), which is characterized by hepatocellular lipid accumulation (steatosis) along with lobular inflammation, hepatocellular ballooning, and fibrosis [2]. NASH is associated with an increased risk of cirrhosis and hepatocellular carcinoma (HCC) [3]. It is also closely associated with metabolic syndrome and type 2 diabetes, suggesting that NASH is the hepatic manifestation of insulin resistance and type 2 diabetes [4]. Thus, establishment of pharmacological treatment for NASH may be a useful strategy to prevent liver cirrhosis and HCC.

A recent study demonstrated that the DPP-4 inhibitor linagliptin alleviates hepatic steatosis and inflammation in a new murine model of NASH with a diabetic background independently of its glucose-lowering effect [5]. It was also reported that sitagliptin, another DPP-4 inhibitor, improves histological changes of NAFLD in mice that have dietary obesity with or without diabetes [6, 7].

Sodium-glucose co-transporter-2 (SGLT2) inhibitors are a new class of oral antidiabetic drugs, which act to reduce hyperglycemia by promoting urinary glucose excretion independently of the secretion or action of insulin [8]. Empagliflozin is a highly selective SGLT2 inhibitor that improves glycemic control in patients with T2DM as monotherapy or when added to metformin, sulfonylurea, or insulin [9]. Moderate weight loss has been a consistent finding in clinical studies of this drug [10]. In diabetic patients, most of the weight loss due to treatment with dapagliflozin was accounted for by fat loss, with significant reductions versus placebo in the volume of both abdominal visceral adipose tissue (VAT) and subcutaneous adipose tissue (SAT) [11]. In a preclinical model of obesity, empagliflozin triggered weight loss associated with reduction of body fat that was probably secondary to calorie wasting in the urine [12].

Based on these reports, we hypothesized that empagliflozin could prevent the development of NASH by acting on hepatic lipid metabolism independently of its glucose-lowering effect and the combination of linagliptin and empagliflozin could improve synergistically. To date, only five studies have examined impacts of SGLT2 inhibitors alone on development of NAFLD and/or NASH in rodent models [13–16]. Although four studies used ipragliflozin and one study used luseogliflozin, all studies demonstrated that SGLT2 inhibitors attenuated development of NAFLD or NASH [13–16]. However, no studies have examined the effect of empagliflozin or the combination of DPP-4 inhibitors and SGLT2 inhibitors on the development of NASH in the presence of diabetes. Therefore, we investigated the effect of empagliflozin and that of linagliptin, a DPP-4 inhibitor, on steatohepatitis and fibrosis in a new mouse model of NASH with a diabetic background, which was generated by administration of streptozotocin (STZ) to C57BL/6J mice at 2 days old combined with a high-fat diet from the age of 4 weeks [17]. We demonstrated that empagliflozin prevented the development of NASH in this model by its anti-inflammatory and anti-fibrotic effects and the combination of empagliflozin and linagliptin improved NASH synergistically with stronger anti-fibrotic effects.

## Methods

### Animals and induction of NASH

The novel mouse model of NASH was generated according to the protocol of Fujii et al. [17] In brief, pregnant C57BL/6 mice were purchased from CLEA Japan (Tokyo, Japan). Neonatal male mice (2 days old) were injected subcutaneously with a low dose of streptozotocin (STZ; 200 μg) and then were fed a high-fat diet (HFD32; CLEA-Japan, Tokyo, Japan) from 4 weeks after birth. This mouse model progresses from NAFLD to NASH by 8 weeks of age and develops HCC at 16 weeks [17]. Male littermates fed a normal diet (CE-2; CLEA-Japan) without injection of STZ were used as a normal control group. All experiments were approved by the animal care and use committee of Dokkyo Medical University.

Both linagliptin and empagliflozin were provided by Boehringer Ingelheim Pharma GmbH Co. (KG, Biberach an der Riss, Germany).

NASH mice aged 6 weeks were divided into the following four groups (n = 6 per group): a vehicle group, a linagliptin (10 mg/kg) group, an empagliflozin (10 mg/kg) group, and a linagliptin (10 mg/kg) + empagliflozin (10 mg/kg) group. The dose of empagliflozin at 10 mg/kg is an effective pharmacological dose used in animal experiments [18].

Six male C57/B16 mice of the same age fed a normal diet were used as the controls. All animals were provided with food and water ad libitum. After grouping, linagliptin and empagliflozin were administered once daily by oral gavage for 3 weeks. Then the mice were sacrificed for collection of liver tissue samples and blood was obtained via cardiac puncture just death. Samples were centrifuged and the serum was stored at −80 °C until use. Fasting blood glucose levels (16 h fast) were measured with a Glutest Neo Sensor (Sanwa Chemical, Shizuoka, Japan). Blood levels of alanine aminotransferase (ALT), triglycerides (TG), free fatty acids (FFA), and glycated albumin (GA) were measured with an auto-analyzer (JEOL Ltd., Tokyo, Japan). Serum insulin was measured with an enzyme immunoassay (Shibayagi Co., Shibukawa, Japan). The TG content of in the liver tissue was measured using a Triglyceride E-test kit (Wako, Tokyo, Japan).

Liver tissue samples from the left lobe was embedded in tissue Tek O.C.T. compound (Sakura Finetek, Tokyo, Japan), snap frozen in liquid nitrogen, and stored at −80 °C. Sections (5 μm) were cut, dried in air, and fixed in acetone. For hematoxylin and eosin (H–E) staining, liver sections prefixed in Bouin’s solution were stained with Lillie-Mayer’s hematoxylin (Muto Pure Chemicals, Tokyo, Japan). Based on observation of H–E stained sections, the NAFLD activity score (NAS) was calculated from the degree of steatosis, lobular inflammation, and hepatocyte ballooning according to the definition of Kleiner et al. [19].

To assess development of liver fibrosis, liver sections were stained with Sirius red. The positive areas of Sirius red staining in five non-overlapping fields were measured with DP manager/controller and WIN Roof Ver.5.8.1 analysis software (Mitani Co., Tokyo, Japan).

Immunohistochemical staining was performed with anti-F4/80 antigen antibody (Spring Bioscience, Pleasanton, CA, USA), anti-α-smooth muscle actin antibody (SMA; Sigma Aldrich, St Louis, MO, USA), and anti-CD26 antibody (Cell Signaling Technology, Beverly, MA, USA). For quantitative analysis of the F4/80-, α-SMA-, and CD26-positive areas, bright images of stained liver section were captured around the central vein at 200× magnification using a digital camera (BX53 Olympus Japan, Tokyo, Japan). Then the positive areas in three to five non-overlapping fields were measured with DP manager/controller and WIN Roof Ver.5.8.1 analysis software.

### Quantitative real-time RT-PCR

Total RNA was extracted from liver tissues using an SV Total RNA Isolation System (Promega, Madison, WI, USA) and was reversed transcribed to obtain cDNA with SuperScript III First Strand Synthesis Super mix (Invitrogen, Carlsbad, CA, USA). Then real-time quantitative RT-PCR was performed using FastStart SYBR Green Master Mix (Roche Applied Science, Mannheim, Germany) and the following probes: type 1 collagen (NM-007742.3), type 3 collagen (NM-009930.2), TNF-α (NM-013693.3), IL-6 (NM_012589.1), MCP-1 (NM-011333.3), SREBP-1c (NM-011480.3), ACC1 (NM-133360.2), ACC2 (NM-133904.2), SCD-1 (NM-009127.4), FAS (NM-134383.2), PPAR-α (NM-001113418.1), ACOX-1 (NM-015729.3), and CD26 (NM-001159543.1).

The expression of mRNAs for TNF-α, IL-6, MCP-1, SOCS3, type 1 collagen, type 3 collagen, FAS, ACC-1, PPAR-α, and ACOX-1 was calculated as a ratio to GAPDH mRNA expression.

### Statistical analysis

Data were expressed as the mean ± standard error of the mean (SEM). Differences between groups were analyzed by the Kruskal–Wallis test with Dunn’s multiple comparison test. A probability (P) value <0.05 was accepted as indicating statistical significance.

## Results

### Effect of empagliflozin and linagliptin on body weight and biochemical parameters

On day 21, body weight was moderately lower in the diabetic animals than in the control group. As expected, plasma glucose was significantly higher in the vehicle group than in the control group and was also significantly higher in the linagliptin group. Serum glycated albumin (GA) was significantly higher in the vehicle group and the linagliptin group than in the control group. Serum GA was lower in the empagliflozin group in compared with the vehicle group or the linagliptin group. Serum ALT was significantly lower in the empagliflozin group than in the vehicle group. Free fatty acids were elevated in the STZ-treated groups compared to the control group. Fasting serum insulin was higher in the linagliptin + empagliflozin group than in the vehicle group (Table 1).

**Table 1:** Body weight and biochemical parameters on day 21 in NASH mice under diabetic background (vehicle, lina 10 mg/Kg, Empa 10 mg/Kg, and Lina+Empa) and their normal littermates

	Control (n = 6)	Vehicle (n = 6)	Lina 10 mg/Kg (n = 6)	Empa 10 mg/Kg (n = 6)	Lina + empa (n = 6)
Body weight (g)					
Day 0	22.6 ± 0.2	20.0 ± 0.9	19.9 ± 0.9	20.3 ± 1.2	20.5 ± 1.0
Day 21	25.1 ± 0.5	21.9 ± 0.7	21.6 ± 0.9*	21.8 ± 1.2	22.8 ± 0.6
Plasma glucose (mg/dl)	184.5 ± 14.1	470.7 ± 53.7^†^	479.0 ± 49.2^†^	319.3 ± 35.1	345.0 ± 57.6
GA (%)	2.35 ± 0.36	5.86 ± 0.73*	5.53 ± 0.43*	2.58 ± 0.50^§, #^	3.32 ± 0.44
ALT (U/l)	28.3 ± 3.7	42.8 ± 5.7	52.5 ± 15.9	20.7 ± 4.0^§^	23.8 ± 2.3
Triglyceride (mg/dl)	40.8 ± 5.3	36.8 ± 7.8	37.3 ± 7.3	26.0 ± 6.3	43.2 ± 8.4
Free fatty acid (mEq/l)	0.76 ± 0.06	1.72 ± 0.15^†^	1.80 ± 0.29^†^	2.02 ± 0.21^‡^	2.10 ± 0.06^‡^
Insulin (ng/ml)	0.53 ± 0.05	0.40 ± 0.07	0.69 ± 0.19	0.79 ± 0.06	0.92 ± 0.18^§^

Data are mean ± SE

*Lina* linagliptin; *Empa* empagliflozin; *GA* glycated albumin; *ALT* alanine aminotransferase

* P < 0.05, ^†^ P < 0.01, ^‡^ P < 0.001 vs. control; ^§^ P < 0.05, ^||^ P < 0.01, ^¶^ P < 0.001 vs. vehicle; ^#^ P < 0.05, ** P < 0.01, ^††^ P < 0.001 vs. linagliptin alone

### Effect of empagliflozin and linagliptin on the liver/body weight ratio and hepatic triglyceride (TG) content

**Fig. 1:**
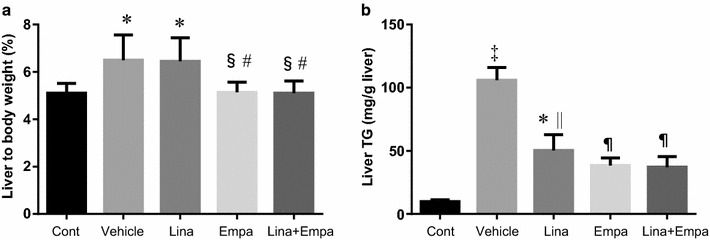
Liver to body weight ratio (**a**) and liver triglyceride content (**b**) in the five groups. Data are mean ± SE. *P < 0.05, ^†^P < 0.01, ^‡^P < 0.001 vs. control; ^§^P < 0.05, ^||^P < 0.01, ^¶^P < 0.001 vs. vehicle; ^#^P < 0.05 vs. Linagliptin alone

The liver/body weight ratio was higher in the vehicle-treated group and the linagliptin-treated group than in the control group, while it was significantly lower in the empagliflozin group and the linagliptin + empagliflozin group than in the vehicle group or the linagliptin group (Fig. 1a). The hepatic TG content was higher in the vehicle group than in the control group, while it was lower in the linagliptin, empagliflozin, and linagliptin + empagliflozin groups compared with the vehicle group (Fig. 1b).

### Effect of empagliflozin and linagliptin on the histological NAFLD activity score (NAS)

**Fig. 2:**
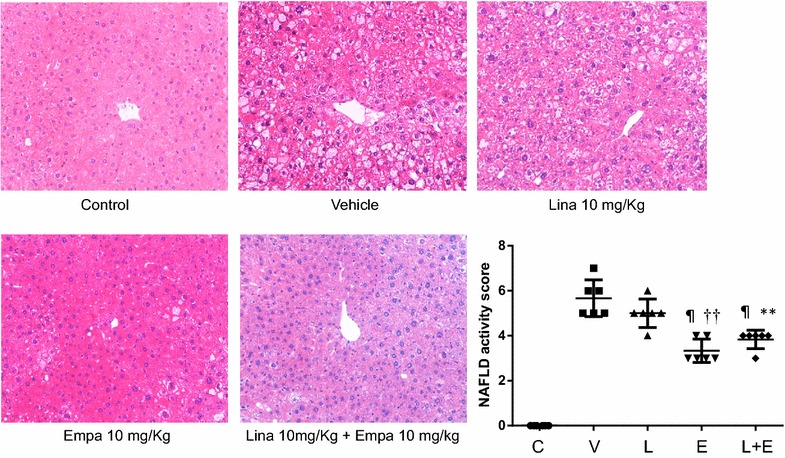
Representative microphotographs of liver sections stained with hematoxylin eosin and NAFLD activity score (*NAS*) in the five groups. Original magnification ×200. Data are mean ± SE. ^¶^P < 0.001 vs. vehicle; **P < 0.01, ^††^P < 0.001 vs. Linagliptin alone

Examination of H–E stained liver sections revealed fatty degeneration, inflammatory cell infiltration, and hepatocellular ballooning, predominantly around the central veins, in mice from the vehicle group. The NAS score was significantly higher in the diabetic animals than in the non-diabetic control group (Fig. 2). The NAS score was significantly lower in the empagliflozin and linagliptin + empagliflozin groups compared with the vehicle group or the linagliptin group. The scores of each component of NAS in all groups were shown in Table 2.

**Table 2:** Nonalcoholic fatty liver disease (NAFLD) activity score in the five groups

	Steatosis	Lobular inflammation	Ballooning	NAS
Control	0	0	0	0
Vehicle	1.3 ± 0.5	2.3 ± 0.5	2	5.7 ± 0.8
Linagliptin 10 mg/Kg	0.8 ± 0.4	2.2 ± 0.4	2	5.0 ± 0.6
Empagliflozin 10 mg/Kg	1	1.3 ± 0.5	1.0 ± 0.6	3.3 ± 0.5
Linagliptin + empagliflozin	0.8 ± 0.4	2	1	3.8 ± 0.4

Data are mean ± SD

*NAS* nonalcoholic fatty liver disease (NAFLD) activity score

### Effect of empagliflozin and linagliptin on hepatic inflammation

**Fig. 3:**
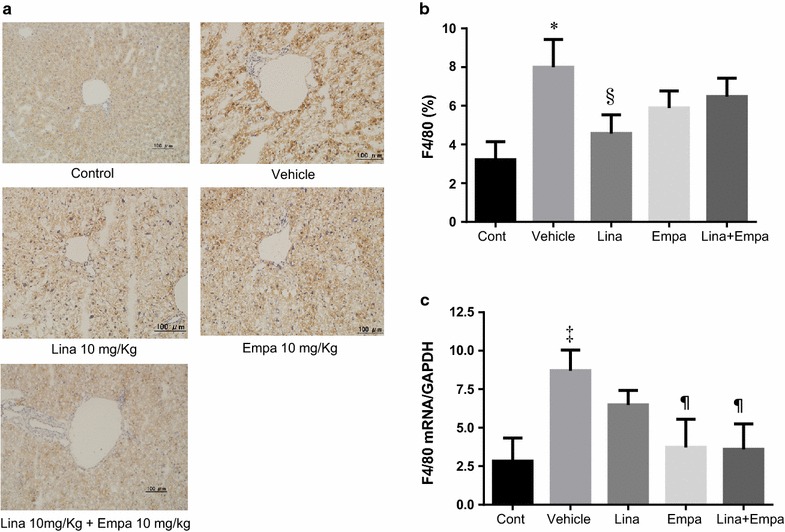
Representative microphotographs of immunohistochemical staining for F4/80 in liver sections (**a**) and percentage in area of positive immunostaining for F4/80 in the five groups (**b**). Normalized mRNA expression of F4/80 the liver of the five groups (**c**). Data are mean ± SE. *P < 0.05, ^‡^P < 0.001 vs. control; ^§^P < 0.05, ^¶^P < 0.001 vs. vehicle

Immunohistochemical staining showed that expression of F4/80 antigen, a marker of macrophages, was increased in the livers of the vehicle-treated mice (Fig. 3a). Treatment with linagliptin significantly reduced F4/80 antigen expression in the peri-central zone of the liver compared with the vehicle group (Fig. 3a). Expression of F4/80 mRNA was increased in vehicle-treated NASH mice compared with control mice, while it was significantly decreased in the empagliflozin and linagliptin + empagliflozin groups compared with the vehicle group (Fig. 3c).

**Fig. 4:**
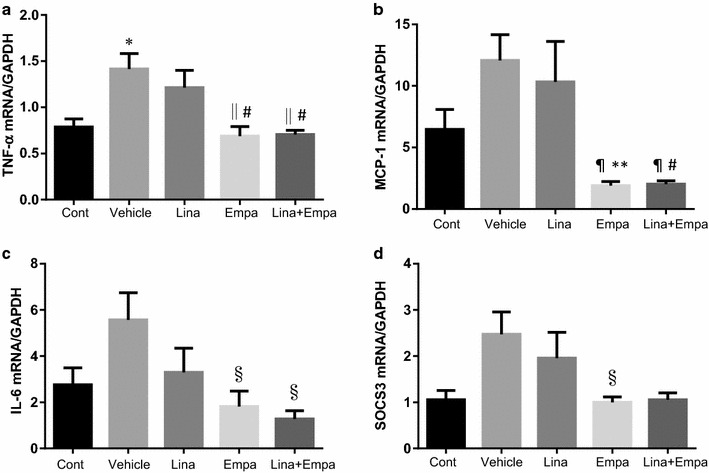
Gene expression of inflammation in the liver of the five groups. Normalized mRNA expression tumor necrosis factor (TNF) α (**a**), monocyte chemoattractant protein (MCP)-1 (**b**), interleukin (IL)-6 (**c**), and suppressor of cytokine signaling (*SOC*)-3 (**d**). Data are mean ± SE. *P < 0.05 vs. control; ^§^P < 0.05, ^||^P < 0.01, ^¶^P < 0.001 vs. vehicle; ^#^P < 0.05, **P < 0.01 vs. Linagliptin alone

Expression of TNF-α mRNA was increased in vehicle-treated NASH mice compared with control mice (Fig. 4), while it was significantly decreased in the empagliflozin and linagliptin + empagliflozin groups compared with the vehicle group or the linagliptin group. Similarly, MCP-1 mRNA expression was significantly decreased in the empagliflozin group and the linagliptin + empagliflozin group relative to the vehicle group or the linagliptin group (Fig. 4). Expression of SOCS3 mRNA was significantly decreased in the empagliflozin group (Fig. 4).

### Effect of empagliflozin and linagliptin on hepatic fibrosis

We next investigated whether empagliflozin prevented the progression of hepatic fibrosis, which is the advanced stage of NASH. First, liver fibrosis was assessed by Sirius red staining.

**Fig. 5:**
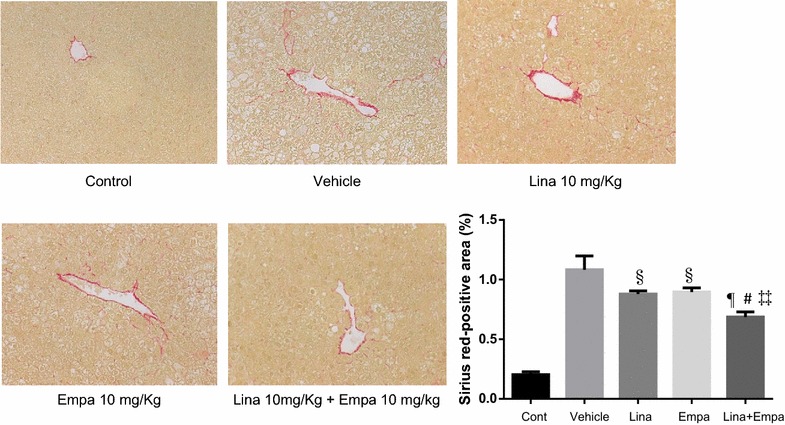
Representative microphotographs of liver sections stained with Sirius red in the liver sections and percentage in area of positive staining for Sirius red in the five groups. Data are mean ± SD. ^§^P < 0.05, ^¶^P < 0.001 vs. vehicle; ^#^P < 0.05 vs. Linagliptin alone; ^‡‡^P < 0.05 vs. Empagliflozin alone

The collagen deposition was significantly lower in the linagliptin group, the empagliflozin group, and the empagliflon + empagliflozin group relative to the vehicle group. In addition, treatment with linagliptin + empagliflozin significantly reduced collagen deposition in the peri-central vein zone of the liver compared with that in the linagliptin or the empagliflozin group (Fig. 5).

**Fig. 6:**
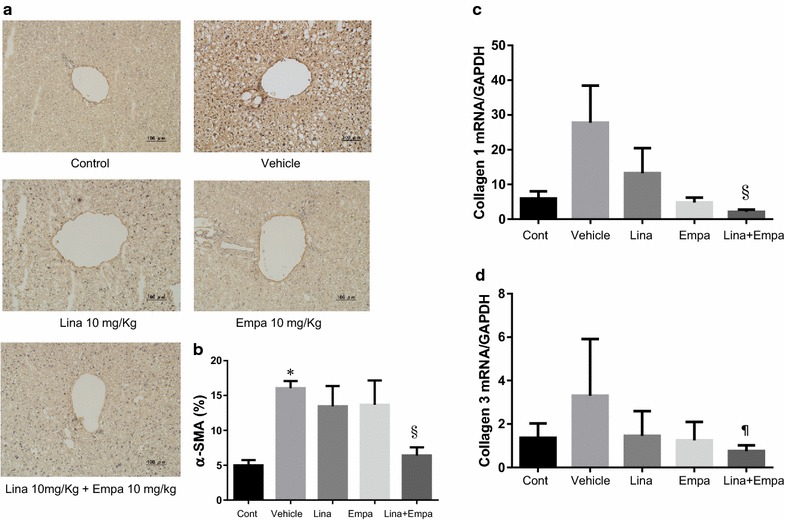
Representative microphotographs of immunohistochemical staining for α-smooth muscle antigen (*SMA*) in liver sections and percentage in area of positive immunostaining for α-SMA in the five groups (**a**, **b**). Gene expression of fibrosis in the liver of the five groups. Normalized mRNA expression of collagen 1 and 3 (**c**, **d**). Data are mean ± SD. *P < 0.05 vs. control; ^§^P < 0.05, ^¶^P < 0.001 vs. vehicle

Immunohistochemical staining showed increased hepatic expression of α-SMA, a marker of fibrosis, in vehicle-treated NASH mice (Fig. 6a, b). Treatment with linagliptin + empagliflozin reduced α-SMA expression in the peri-central vein zone of the liver compared with that in the vehicle group. In addition, expression of both type 1 and 3 collagen mRNA was significantly lower in the linagliptin + empagliflozin group than in the vehicle group (Fig. 6c, d).

### Effect of empagliflozin and linagliptin on hepatic fat metabolism

**Fig. 7:**
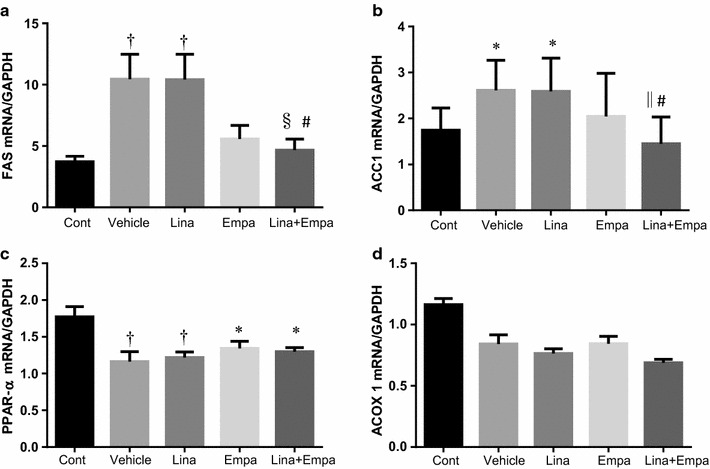
Gene expression of lipid metabolism in the liver of the five groups. Normalized mRNA expression of fatty acid synthase (*FAS*) (**a**), acetyl-CoA carboxylase (*ACC*) 1 (**b**), peroxisome proliferator-activated receptor (PPAR) α (**c**), and acyl-coenzyme A oxidase (ACOX)1 (**d**). Data are mean ± SD. *P < 0.05, ^†^P < 0.01, ^‡^P < 0.001 vs. control; ^§^P < 0.05, ^||^P < 0.01, ^¶^P < 0.001 vs. vehicle; ^#^P < 0.05, **P < 0.01, ^††^P < 0.001 vs. Linagliptin alone

We subsequently investigated whether empagliflozin had an influence on lipid metabolism in the liver. Expression of mRNA for FAS, a gene involved in fatty acid production (lipogenesis), was significantly higher in vehicle-treated NASH mice than in control mice. The expression of FAS mRNA was significantly lower in the linagliptin + empagliflozin group than in the vehicle group or the linagliptin group. Similarly, expression of mRNA for ACC1, another gene involved in lipogenesis, was significantly lower in the linagliptin + empagliflozin group than in the vehicle group or the linagliptin group (Fig. 7). We found no significant differences in the expression of beta oxidation-related genes in the five groups.

On the other hand, the expression of mRNA for PPAR-α was significantly decreased in the NASH groups compared with the control group irrespective of drug treatment. Neither linagliptin nor empagliflozin affected the expression of PPAR-α and ACOX1, both genes involved in β-oxidation (fatty acid oxidation), in NASH mice with diabetes.

### Effect of empagliflozin and linagliptin on hepatic CD26/DPP-4 expression

Since plasma DPP-4 activity is increased in patients with NAFLD [20] and patients who have type 2 diabetes with elevated liver enzymes [21], treatment with DPP-4 inhibitors may prevent the development of NASH. Therefore, we investigated the effects of linagliptin or empagliflozin on expression of CD26/DPP-4 in the liver. Immunohistochemical staining showed that CD26/DPP-4 expression was unexpectedly higher in the linagliptin group than in the vehicle group (data not shown). Hepatic expression of CD26 mRNA was significantly lower in the vehicle, linagliptin, empagliflozin, and linagliptin + empagliflozin groups than in the control group (data not shown).

## Discussion

The present study demonstrated for the first time that the combination of empagliflozin and linagliptin prevented development of NASH in a novel mouse model of NASH with diabetes. NAS was significantly prevented in both the animals treated with empagliflozin alone and those receiving linagliptin + empagliflozin compared with the vehicle group or the linagliptin group. Treatment with empagliflozin ameliorated hepatic steatosis, inflammation, and fibrosis in this novel model of NASH and diabetes. We obtained histological proof that empagliflozin prevents development of NASH in our mouse model of NASH and diabetes. Several studies have shown that other SGLT2 inhibitors (ipragliflozin and luseogliflozin) alleviate hepatic steatosis or steatohepatitis in obese type 2 diabetic mice or rats [13–16]. However, all other studies examined the effects of SGLT2 inhibitors alone on development of NASH in a variety of rodent models [13–16]. The present study demonstrated for the first time that the combination of SGLT2 inhibitor and DPP-4 inhibitor can synergistically ameliorate NASH with stronger anti-fibrotic effects compared with each drug alone in a new model of NASH and diabetes, because we found that the extent of liver fibrosis evaluated by Sirius red and alpha-SMA staining was significantly smaller in the combination of empagliflozin and linagliptin group than in the empagliflozin group or the linagliptin group.

Treatments with empagliflozin alone as well as linagliptin alone decreased the hepatic TG content, in agreement with the findings of a previous study regarding linagliptin [5]. The TG content of the liver is closely associated with steatosis in NASH as well as with de novo lipogenesis [22]. Thus, empagliflozin could be a novel therapy for both NAFLD and NASH associated with diabetes. The mechanisms underlying reduction of the hepatic TG content by empagliflozin remain to be elucidated. One possible explanation is that empagliflozin inhibits an increase of de novo lipogenesis, since the present study showed that expression of both FAS and ACC1, genes involved in de novo lipogenesis [23], was significantly lower in the linagliptin + empagliflozin group than in the vehicle group or the linagliptin group. FAS is a key enzyme involve in hepatic fatty acid biosynthesis and is believed to be a determinant of the maximal capacity of the liver to synthesize fatty acids by de novo lipogenesis, because it catalyzes the last step in the fatty acid biosynthetic pathway [23]. Thus, improvement of NASH by empagliflozin may be associated with a decrease of fatty acid production (de novo lipogenesis) rather than an increase of fatty acid oxidation. In fact, a very recent study also has demonstrated that ipragliflozin improved hepatic steatosis in high-fat-induced and leptin-deficient (ob/ob) obese irrespective of body weight reduction, and that ipragliflozin reduced the gene expression of de novo lipogenesis, while it did not change the expression of β oxidation-related genes [15].

Inhibition of SGLT2 has been shown to reduce glucose reabsorption, which means substantial urinary loss of glucose (calories) in adult patients taking SGLT2 inhibitors [8, 10]. Over a certain term, the cumulative loss of calories may lead to substantial weight loss [8, 10]. A meta-analysis demonstrated that ≥5 % weight loss improves hepatic steatosis and ≥7 % weight loss also improves NAS, although fibrosis was unchanged [24]. However, we found no significant difference in body weight among the five groups after treatment, suggesting that improvement of NASH by empagliflozin may be independent of weight reduction. Mechanisms responsible for the lack of weight reduction by empagliflozin in our study remain to be unclear. A previous study reported that the persistent urinary glucose excretion induced by dapagliflozin was accompanied by compensatory hyperphagia, which attenuated the weight loss induced by SGLT2 inhibition [25]. Because all animals were provided with food and water ad libitum in our study, it is possible that overeating induced by empagliflozin was associated with lack of effects on weight loss in the empagliflozin group or in the combination of empagliflozin and linagliptin group.

Accumulation of fat in the liver can occur due to excess dietary intake, increased delivery of fatty acid to the liver, inadequate fatty acid oxidation, and increased de novo lipogenesis [17]. Hepatic FAS synthesizes lipids (fatty acids) that are stored as lipid droplets [26]. Thus, reduction of hepatic FAS (and ACC1) mRNA levels by empagliflozin may have contributed to the improvement of NASH independently of weight reduction in our mouse model.

The present study also demonstrated for the first time that the combination of empagliflozin and linagliptin reduced the collagen deposition (Sirius red staining areas) and the expression of α-SMA protein, a marker of activated myofibroblasts (hepatic stellate cells) [27], around the central veins, as well as decreasing the expression of type 1 and 3 collagen mRNA. It has been suggested that α-SMA may be a valuable marker for evaluation of stellate cell activation and progression of hepatic fibrosis [27]. If this combination therapy can prevent hepatic fibrosis, an advanced stage of NASH, it could subsequently stop progression to cirrhosis and HCC in NASH patients with diabetes.

The mechanisms contributing to reduction of hepatic fibrosis by the combination of empagliflozin and linagliptin remain unclear. One possible explanation for the anti-fibrotic effect of this combination therapy is that linagliptin prevents the infiltration of macrophages (F4/80 positive cells) into lobular lesions, while empagliflozin inhibits the hepatic expression of proinflammatory cytokines such as IL-6, TNF-α, and MCP-1, which are regarded as hallmarks of NASH [28]. Thus, inhibition of inflammation in the liver may contribute to inhibition of hepatic fibrosis in NASH mice treated with the combination of empagliflozin and linagliptin.

A number of drugs have the potential to alleviate NASH in diverse ways, such as anti-inflammatory and antioxidant actions, reduction of de novo lipogenesis, and promotion of β-oxidation [29]. Several studies have demonstrated that DPP-4 inhibitors can prevent and improve liver steatosis in diabetic mice or rodents fed a high-fat diet [6, 7]. In the same model as ours, linagliptin alleviates hepatic steatosis and inflammation by reducing macrophages infiltration and lobular inflammation, as well as expression of TNF-α and IL-6 [30]. The improvement of hepatic steatosis by linagliptin was also accompanied by a decrease in expression of fatty acid synthase [5, 30]. In the present study, we failed to find a significant reduction of NAS in the linagliptin group. Both the empagliflozin and linagliptin + empagliflozin groups showed significant improvements of NAS and reduction of gene expression of proinflammatory cytokines in the liver compared with the vehicle or linagliptin groups, suggesting that empagliflozin may have a stronger effect on NASH than linagliptin in this model.

We found that empagliflozin significantly decreased the expression of SOCS3 mRNA in the livers of NASH mice compared with vehicle-treated NASH mice. Expression of IL-6 mRNA was also significantly decreased in the empagliflozin group and the linagliptin + empagliflozin group relative to that in the vehicle group. IL-6 blocks the insulin-signaling pathway, partly by inducing SOCS3 in hepatocytes [31]. SOCS3 inhibits the action of insulin by biding to the insulin receptor or IRS-1 and-2, followed by their ubiquitination and degradation [32]. Although it was reported that linagliptin inhibits hepatic SOCS3 expression in NAFLD and NASH mice [5, 30], we failed to find a significant reduction of SOCS3 mRNA expression in the liver after treatment with linagliptin. Thus, empagliflozin may have the potential to ameliorate insulin resistance in the liver due to inhibition of the IL-6-SOCS3 axis.

The present study had some limitations. Because of a significant difference in plasma glucose level after treatment between empagliflozin and linagliptin, the possibility that empagliflozin may prevent development of NASH partly due to its glucose-lowering effects could be not excluded in this study. Another limitation is that this model lacks obesity and insulin resistance, which are associated with NASH in humans.

In conclusion, we demonstrated that empagliflozin, an SGLT2 inhibitor, has the potential to prevent NASH through its anti-steatotic and anti-inflammatory actions in a mouse model of NASH with diabetes. In addition, the combination of empagliflozin and linagliptin synergistically prevented development of liver fibrosis in this model. Thus, empagliflozin prevented development of the pathological features of NASH, suggesting that it could become a simple new therapeutic strategy for NASH associated with diabetes.
